# Retrospective Study of Complicated Pneumonia at the Pediatric Department of the University Hospital of Padua: Experience from 2022 to 2024

**DOI:** 10.3390/jcm15030978

**Published:** 2026-01-26

**Authors:** Valentina Agnese Ferraro, Fiorenza Alfier, Giulia Brigadoi, Daniele Donà, Luca Marchetto, Benedetta Marino, Alberto Sgrò, Federica Visentin, Andrea Volpe, Stefania Zanconato, Silvia Carraro

**Affiliations:** 1 Women’s and Children’s Health Department–SDB, University of Padua, 35128 Padova, Italy; alfier.fiorenza@gmail.com (F.A.); giulia.brigadoi@unipd.it (G.B.); daniele.dona@unipd.it (D.D.); silvia.carraro@unipd.it (S.C.); 2 Unit of Pediatric Allergy and Respiratory Medicine, Women’s and Children’s Health Department, University-Hospital of Padova, 35128 Padova, Italy; stefania.zanconato@aopd.veneto.it; 3 Division of Pediatric Infectious Diseases, Department of Women’s and Children’s Health, University-Hospital of Padua, 35128 Padova, Italy; 4 Pediatric Intensive Care Unit, Department of Women’s and Children’s Health, University-Hospital of Padova, 35128 Padova, Italy; luca.marchetto@aopd.veneto.it; 5 Pediatric Surgery Unit, Department of Women’s and Children’s Health, University-Hospital of Padova, 35128 Padova, Italy; benedetta.marino22@gmail.com (B.M.); alberto.sgro@aopd.veneto.it (A.S.); andrea.volpe@aopd.veneto.it (A.V.); 6 Pediatric Emergency Division, Department of Women’s and Children’s Health, University-Hospital of Padova, 35128 Padova, Italy; federica.visentin@aopd.veneto.it

**Keywords:** complicated community-acquired pneumonia, children, necrotizing pneumoniae

## Abstract

**Background:** Community-acquired pneumonia (CAP) in children may be complicated by necrotizing pneumonia (NP), complicated parapneumonic effusion (CPPE), and lung abscess. These complications prolong hospitalization and require medical and surgical intervention. Objectives. To describe clinical course, diagnostic workup, and management of cCAP (complicated CAP) in children admitted to the Women’s and Children’s Health Department, Padua University Hospital, between January 2022 and September 2024. To identify factors associated with disease severity and evaluate outcomes. **Methods:** All children hospitalized for cCAP during the study period were included. Data collected comprised clinical features, laboratory and imaging findings, medical and surgical management, and outcomes. **Results:** Forty patients (mean age 4.4 y; 13.15% of pneumonia admission) were included: 67.5% had NP with CPPE, 22.5% isolated effusion, 10% NP without effusion. All patients were febrile at onset, 62.2% had cough, 32.5% abdominal pain, 30% rhinitis. NP was confirmed by contrast-enhanced chest CT. Thirty patients (75%) had positive microbiological testing, mainly *Streptococcus pneumoniae* and *Streptococcus pyogenes*. 77.5% required oxygen therapy (five invasive ventilation and one with ECMO). Median fever duration 18 days (IQR 15–27) with elevated CRP (median peak 300 mg/L). Pleural drainage was performed in 66.7%, fibrinolytics in 17.5%, thoracoscopic decortication in 12.5%, and lobectomy in one patient. Radiological resolution occurred at a median of 31 days post-discharge, with normal pulmonary function at a median of 15 months. **Conclusions:** Despite pediatric cCAP severity, short- and long-term outcomes are favorable. Early recognition and integrated management are crucial, and further prospective studies are warranted to optimize care and identify severity predictors.

## 1. Introduction

Community-acquired pneumonia (CAP) represents one of the leading causes of morbidity and hospitalization among children worldwide [1]. Although the majority of cases respond favorably to prompt and appropriate antibiotic therapy, a subset of patients develops complicated community-acquired pneumonia (cCAP), a severe clinical condition that should be suspected in children who fail to show clinical improvement within 48–72 h after treatment initiation [2]. cCAP includes necrotizing pneumonia (NP), empyema and complicated parapneumonic effusion (CPPE), and lung abscess [2].

NP is a severe complication of CAP characterized by progressive destruction of the pulmonary parenchyma with the development of necrosis despite adequate antibiotic therapy [3,4]. This process evolves rapidly, leading to cavitation and loss of lung tissue, and is frequently associated with empyema and, in some cases, the development of bronchopleural fistulae [5]. NP was first described in the pediatric population in 1994 [6], and, although relatively uncommon, its incidence has been increasing. It is estimated to complicate approximately 0.8–7% of all CAP cases [2,6,7,8]. The mean age at presentation ranges between 2 and 5 years, with no significant gender difference, and affected children typically have no underlying comorbidities [4,9]. Clinically, children with NP typically present with persistent fever and respiratory distress [10]. The principal causative pathogens are *Streptococcus pneumoniae* and *Staphylococcus aureus*, although other pathogens including *Streptococcus pyogenes*, *Haemophilus influenzae*, and *Mycoplasma pneumoniae*, have also been reported [3,10]. Laboratory findings frequently reveal marked leukocytosis, elevated C-reactive protein, anemia, and hypoalbuminemia, while pleural fluid, when present, commonly shows features of empyema [10]. Imaging plays a pivotal role in diagnosis, with contrast-enhanced chest computed tomography (CT) representing the gold standard for identifying necrotic areas within consolidated lung parenchyma [6,11]. The management of NP and other forms of cCAP remains challenging and requires a multidisciplinary approach involving pediatric pulmonologists, intensive care physicians, infectious disease physicians, and thoracic surgeons. Children with NP generally require prolonged intravenous antibiotic therapy, with a mean duration of approximately 28 days [7,8], followed by an additional 10–14 days of oral antibiotic once sustained clinical and laboratory improvement has been achieved [12]. In selected cases, surgical intervention may be necessary for the drainage of large pleural effusions or hydropneumothorax [10,11], often in conjunction with intrapleural fibrinolytic therapy (e.g., urokinase or tissue plasminogen activator). In a minority of patients, video-assisted thoracic surgery (VATS) with segmental or lobar lung resection may be required [13,14]. Although NP is associated with prolonged hospitalization and invasive procedures, most children demonstrate normal pulmonary function and near-complete radiological resolution within 1–3 months following discharge [3].

CPPE is defined as the abnormal accumulation of fluid within the pleural space, which is bounded by the visceral and parietal pleura [4]. In the pediatric population, parapneumonic effusion occurs in approximately 13–28% of hospitalized children with cCAP [7]. Its pathogenesis is driven by inflammation-induced rises in capillary permeability and typically progresses through three stages: exudative, fibrinopurulent, and organized empyema [15,16]. *S. pneumoniae* remains the predominant etiology, followed by *S. aureus* and *S. pyogenes*; however, a broader spectrum of bacterial pathogens, and less frequently viral agents or *Mycobacterium tuberculosis*, may also be involved [15]. Clinical manifestations include persistent fever, chest or abdominal pain, and reduced breath sounds. Thoracic ultrasound is considered the imaging modality of choice for both diagnosis and procedural guidance [17]. Management consists of supportive care, appropriate antibiotic therapy, and pleural drainage when indicated. Intrapleural fibrinolysis has proven effective in the treatment of loculated effusions and may reduce the need for surgical intervention, although VATS remains necessary in refractory cases [18]. Overall prognosis is excellent, with most children achieving complete recovery [18].

The primary aim of this study is to describe the clinical course, diagnostic workup, and therapeutic strategies in children with cCAP hospitalized at the Department of Women’s and Children’s Health, University Hospital of Padua, between January 2022 and September 2024. Secondary aims include the identification of factors potentially associated with a more severe disease course and the assessment of short- and long-term functional and radiological outcomes following cCAP.

## 2. Materials and Methods

### 2.1. Study Population

This is a retrospective observational study carried out at the Department of Women’s and Children’s Health, University Hospital of Padua. All patients aged 0–18 years who were hospitalized with a diagnosis of cCAP between January 2022 and September 2024 were eligible for inclusion. The study population comprised all patients who fulfilled predefined diagnostic criteria for cCAP during the enrollment period. cCAP was defined by the presence of at least one of the following conditions: necrotizing pneumonia (NP), complicated parapneumonic effusion (CPPE), or lung abscess. NP was diagnosed on the basis of radiological evidence of parenchymal necrosis, characterized by areas of low attenuation, cavitation, or loss of normal lung architecture on CT. CPPE was defined by the presence of pleural fluid associated with pneumonia requiring invasive drainage and/or demonstrating biochemical, microbiological, or radiological features consistent with empyema, including loculations or pleural thickening. Lung abscess was diagnosed in the presence of a well-defined intraparenchymal cavitary lesion containing air–fluid levels, as documented by chest radiography or CT. All patients meeting these diagnostic criteria during the study period were included without additional selection based on clinical severity, management strategy, or outcome. Data were retrospectively retrieved from electronic medical records. Patients admitted for non-infectious conditions or for infections other than cCAP were excluded.

The study was approved by the Ethics Committee of the University Hospital of Padua (approval number AOP3263). Written informed consent was obtained from parents or legal guardians of all minor participants, and directly from patients when of legal age, in accordance with Italian legislation on data protection and research ethics.

### 2.2. Data Collection

For each patient, demographic characteristics and clinical history were recorded, including age, sex, weight, height, vaccination status, comorbidities, and previous respiratory conditions, with particular attention to recurrent respiratory infections and prior episodes of pneumonia. Data regarding clinical presentation were collected, encompassing presenting symptoms, physical examination findings, and timing of symptom onset. Laboratory investigations performed at hospital admission and throughout hospitalization were collected and included complete blood count, inflammatory markers (C-reactive protein, procalcitonin), and biochemical profile. Microbiological assessments comprised blood cultures, pleural fluid cultures when available, and molecular testing for viral and bacterial pathogens on nasopharyngeal aspirates or nasal swabs. All samples were obtained following standardized clinical procedures. Imaging data, including chest X-ray and contrast-enhanced chest CT, were also systemically collected and analyzed.

According to hospital protocol, blood cultures (with antibiogram) were routinely obtained in all patients (before starting intravenous antibiotic therapy). Pleural fluid cultures (with antibiogram) were performed in patients undergoing chest drainage or thoracentesis. Respiratory virus identification was performed using PCR assays on nasopharyngeal aspirates or nasal swabs, as part of routine clinical diagnostic testing in our hospital microbiology laboratory (according to standard laboratory protocols).

Data on therapeutic strategies were also collected, including medical management, such as supportive care and antibiotic therapy, as well as surgical interventions, namely chest tube drainage, intrapleural fibrinolysis, and video-assisted thoracic surgery [VATS], with or without decortication or localized pulmonary resection. Additional data included length of hospital stay and clinical, radiological, and functional outcomes assessed at both short- and long-term follow-up.

Pulmonary function was assessed with a 10 L bell spirometer (Biomedin, Padua, Italy). At least three spirometric maneuvers were performed, with two reproducible curves required for test validity. Forced vital capacity (FVC) and forced expiratory volume in one second (FEV1) were analyzed, using the most accurate values among the three maneuvers. Spirometric values were expressed as Z-scores based on the Global Lung Function Initiative reference equations endorsed by the European Respiratory Society [19,20].

### 2.3. Statistical Analysis

Data were stored and processed using Microsoft Excel. Descriptive statistics were employed to summarize the characteristics and distribution of clinical variables. Data were presented as absolute number (n) and percentages (%), means with standard deviations or medians with interquartile ranges (IQR), as appropriate. Variations in antibiotic therapy over time for each patient were visualized using Sankey diagrams generated with R-Studio, with link widths proportional to the magnitude of treatment flows. Associations between categorical variables were assessed using Fisher’s exact test. To identify potential factors associated with cCAP severity, multiple linear regression analysis was performed, with fever duration as the dependent variable and age, presence of systemic complications (hypoalbuminemia, hyponatremia), number of lobes involved, presence of necrotizing pneumonia, and C-reactive protein (CRP) values (< or >300 mg/L) as independent variables. A *p*-value < 0.05 was considered statistically significant.

## 3. Results

**Figure 1:**
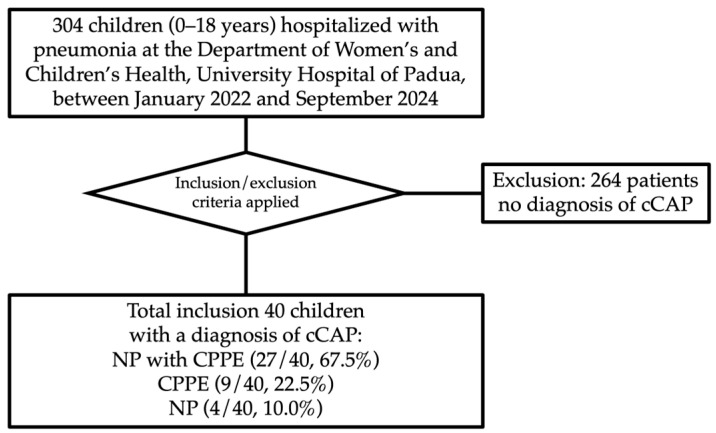
Flow diagram illustrating the patient selection process for the study cohort. cCAP = complicated community acquired pneumonia; NP = necrotizing pneumonia; CPPE = complicated parapneumonic effusion.

Over the study period at the Department of Women’s and Children’s Health of the University Hospital of Padua, 304 children were hospitalized with pneumonia, of whom 40 (13.15%) had cCAP (Figure 1).

Among these, 26/40 patients (65.0%) had been referred from other hospitals in the North East of Italy, while 14/40 (35.0%) were admitted directly to our center. Half of the patients (20/40) were female. The mean age at presentation was 4.4 years (SD 2.6). The age distribution of the study population is summarized as follows: five patients (12.5%) were younger than 2 years of age, while the largest subgroup consisted of 15 patients (37.5%) aged between 2 and 3 years. Eleven patients (27.5%) were in the 4–5-year age range. Six patients (15.0%) were aged between 6 and 7 years, and the remaining three patients (7.5%) were older than 8 years. Overall, the cohort was predominantly composed of young children, with the majority of participants being under 6 years of age.

The most frequent form of cCAP was NP with CPPE (27/40, 67.5%), followed by isolated CPPE (9/40, 22.5%) and NP without effusion (4/40, 10.0%) (Figure 1). No cases of lung abscess were observed. Clinical complications included pneumatocele in 7 patients (17.5%), hydropneumothorax in 10 (25.0%), and bronchopleural fistula in 5 (12.5%). The right lung was involved in 21 cases (52.5%), the left lung in 16 (40.0%), and both in 3 (7.5%).

Most patients (34/40, 85%) had no relevant past medical history for respiratory problems. Six patients (15%) had comorbidities, including: bilateral renal pyelectasia; acute myeloid leukemia (FAB M2, t(8;21)) under induction therapy; ANA-negative, RF-negative, HLA-B27-negative polyarticular juvenile idiopathic arthritis treated with methotrexate and tocilizumab; history of meningosepsis (two years prior to pneumonia diagnosis); obesity; autism spectrum disorder.

All patients had been vaccinated according to the national immunization schedule, including the hexavalent vaccine (diphtheria, tetanus, acellular pertussis, poliomyelitis, *Haemophilus influenzae* type b, and hepatitis B) and the 13-valent pneumococcal conjugate vaccine (PCV13).

After the cCAP, 10 patients (25%) underwent immunological evaluation, including complete blood count, serum immunoglobulin classes (IgA, IgM, IgG), and anti-tetanus antibody titers: all results were within the normal range

### 3.1. Clinical Presentation

At the time of clinical onset, all patients were febrile. Beside fever, most patients presented with cough (62.5%), rhinitis (30%), chest pain (17.5%), and dyspnea (12.5%). Gastrointestinal symptoms were also frequent, with 32.5% of patients reporting abdominal pain and 20.0% vomiting. Systemic symptoms included anorexia in 22.5%, fatigue in 10.0%, and skin rash in 10.0% of patients. The clinical course was characterized by prolonged fever, with a median duration of 18 days (IQR 15–27), and persistence or worsening of initial symptoms despite appropriate antibiotic therapy. These included cough with tachypnoea, chest pain, fatigue and anorexia. Parenteral nutrition was required in 40% of cases.

On chest auscultation, all patients presented with abnormal findings, including diminished vesicular breath sounds, rales, crackles, and bronchial breath sounds. Oxygen saturation was decreased in 67.5% of cases, necessitating supplemental oxygen therapy.

Severe systemic complications at disease onset were uncommon and were observed in two patients (5.0%). One patient presented with clinical features consistent with septic shock, associated with syndrome of inappropriate antidiuretic hormone secretion (SIADH) and microhematuria, while the other developed acute kidney injury.

### 3.2. Laboratory Findings

All patients demonstrated markedly elevated inflammatory markers. CRP reached a median peak of 300 mg/L (IQR 204.5–388) at a median of 6 days from symptom onset (IQR 5–9.5), while the median peak value for procalcitonin (PCT) was 14.39 µg/L (IQR 2.72–48.4). Interestingly, peak CRP and PCT levels did not correspond: at the time of maximum CRP, median PCT was 4.64 µg/L (IQR 2.20–21.52).

Hematologic abnormalities were common. All patients had neutrophilic leucocytosis and thrombocytosis, and 92.5% (37/40) developed anemia, with nearly half (48.6%) required at least one red blood cell transfusion. Hyponatremia (serum sodium < 136 mmol/L) occurred in 55% of patients. 25 patients (62.5%) required albumin supplementation.

### 3.3. Microbiological Findings

**Figure 2:**
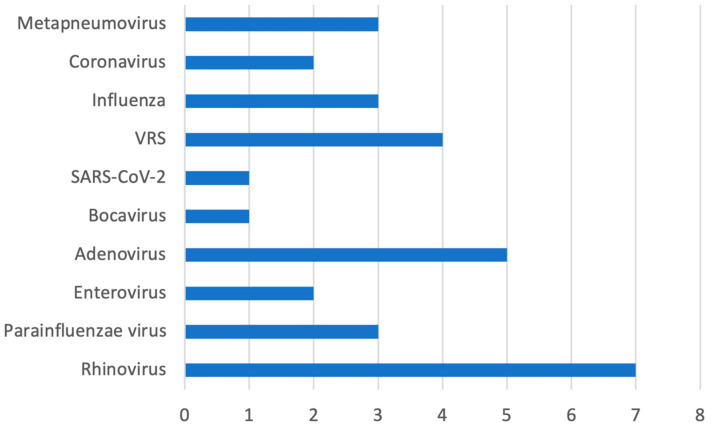
The bar chart shows the distribution of viral positivity detected in throat swabs or nasopharyngeal aspirates.

Microbiological tests (blood culture, pleural fluid culture, and/or nasopharyngeal swab/aspirate) were negative in 25% of children (10/40). Among the remaining patients, microbiological investigations yielded positive results in a subset of cases. Blood cultures were positive in 7 out of 40 patients (17.5%), with *S. pnuemoniae* identified in 5 cases, *S. pyogenes* in one case and *S. aureus* in one case. Pleural fluid cultures were positive in 5 out of 40 patients (12.5%), all of which yielded *S. pyogenes*. Viral nucleic acid detection by PCR on nasopharyngeal aspirate was positive in 15 out of 20 tested patients (75.0%), while PCR performed on throat swabs was positive in 4 out of 20 tested patients (20.0%), resulting in an overall viral positivity in 19 out of 40 patients (47.5%) (Figure 2). Concomitant bacterial and viral infections were identified in 4 out of 40 patients (10.0%)—specifically, two bacterial cultures positive in pleural fluid and two in blood, each with concurrent viral detection in nasopharyngeal aspirate.

Blood cultures were obtained from all patients upon arrival at the Emergency Department, prior to initiation of intravenous antibiotic therapy. However, 10/40 children (25.0%) had already received oral antibiotics before blood sampling. None of the children treated at home had positive blood cultures. Among the remaining 30 patients (75.0%) who had not previously received antibiotics, 7 (23%) had positive cultures, while 23 (76.6%) were negative. Comparison between the two groups (children treated with oral antibiotics at home versus untreated) revealed no statistically significant difference in blood culture positivity (Fisher’s exact test, *p* = 0.1612).

### 3.4. Imaging Studies

The diagnosis of cCAP was established using contrast-enhanced chest CT in 34 of 40 patients (85.0%) and chest radiography in the remaining 6 patients (15.0%). In all patients diagnosed with NP (31/40, 77.5%), contrast-enhanced chest CT was used to confirm the diagnosis. Because of the prolonged disease course, imaging examinations were repeated multiple times during hospitalization. The median number of chest radiographs per patient was 5 (IQR 3.75–10), with a maximum of 41 examinations in a single patient. Chest CT, with or without contrast enhancement, was performed at least once in 34 of 40 patients (85%), with two or more CT scans conducted in 10 patients (25.0%) and up to four CT examinations in a single case. Indications for repeat chest X-ray and/or chest CT included clinical criteria such as persistence or worsening of symptoms (e.g., fever, dyspnea), lack of clinical improvement, increased oxygen requirement, or respiratory deterioration. In addition, laboratory findings suggestive of ongoing or worsening inflammation (e.g., persistently elevated inflammatory markers) prompted further imaging in selected cases. Chest X-rays were also performed for postoperative monitoring in patients who underwent surgical procedures, in accordance with standard clinical protocols.

### 3.5. Factors Associated with cCAP Greater Severity

**Figure 3:**
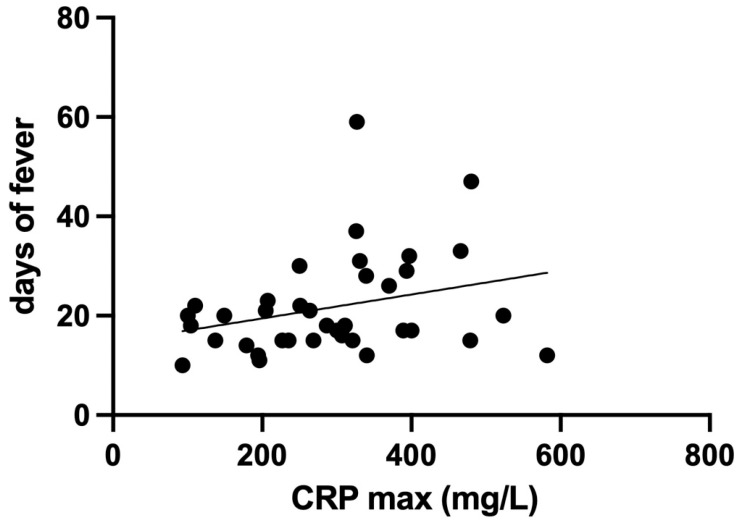
The scatter plot shows the distribution of each patient according to their maximum CRP value (*x*-axis) and days of fever (*y*-axis). Each point represents an individual subject. The line indicates the trend toward significance (*p* = 0.06).

To explore factors potentially associated with a more severe course of cCAP, a multiple linear regression analysis has been performed using fever duration as the dependent variable. Independent variables included age, presence of systemic complications (hypoalbuminemia, hyponatremia), number of affected lung lobes, presence of necrotizing pneumonia, and CRP level (< or >300 mg/L). The analysis showed only a trend toward significance for CRP (*p* = 0.06). Similar results were observed analyzing the correlation between CRP, as a continuous variable, and fever duration (R = 0.3 *p* = 0.07) (Figure 3).

### 3.6. Therapeutic Approach

#### 3.6.1. Antibiotic Therapy

**Figure 4:**
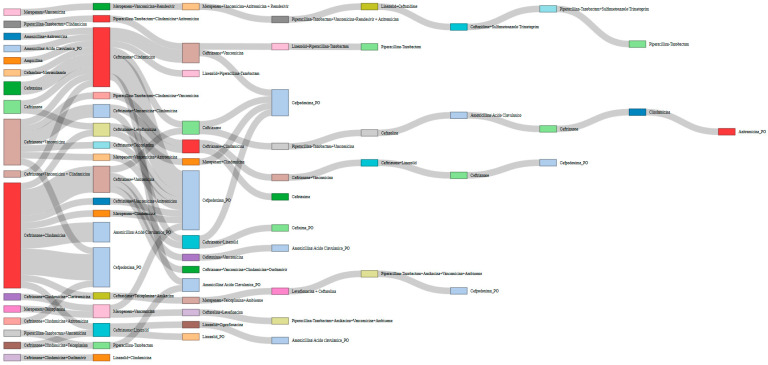
Sankey diagram illustrating modifications in antibiotic therapy during hospitalization. Each flow represents a transition from an initial antibiotic regimen to a subsequent one, with flow thickness proportional to the number of patients undergoing each change. The most common first-line therapy was ceftriaxone plus clindamycin (16/40 patients, 40.0%), followed by ceftriaxone plus vancomycin (7/40, 17.5%). Second-line intravenous therapy was administered in 31 of 40 patients (77.5%), third-line therapy in 18 (45.0%), while further adjustments—up to an eighth-line regimen—were required in a small minority of cases (7/40, 17.5% who required fourth-line therapy or beyond). Overall, antibiotic therapy was modified once in 14 patients (35.0%), two to three times in 9 (22.5%), four to five times in 5 (12.5%), and more than five times in 3 (7.5%). ‘_PO’ indicates oral therapy prescribed at discharge.

Among the 40 children with cCAP included in the study, 10 out of 40 (25.0%) had received oral antibiotics before Emergency Department presentation (amoxicillin in 6 cases, amoxicillin–clavulanate in 3, cefpodoxime in 1). During hospitalization, prolonged intravenous antibiotic therapy was required, with a median duration of 18 days (IQR 16–25.5). In 9 of 40 patients (22.5%), the initial regimen was continued, whereas the remaining 31 patients (77.5%) required at least one modification, i.e., the discontinuation of the ongoing antibiotic regimen and the initiation of a different antibiotic therapy. Treatment modification was undertaken in cases of clinical, laboratory, and/or radiological deterioration, or following microbiological test results showing pathogen positivity with corresponding antibiogram findings. The most common first-line therapy was ceftriaxone plus clindamycin (16/40, 40.0%), followed by ceftriaxone plus vancomycin (7/40, 17.5%). Second-line intravenous therapy was administered in 31 of 40 patients (77.5%), third-line in 18 (45.0%), and further adjustments—up to an eighth-line regimen—were required in a small minority (7/40, 17.5% who required fourth-line therapy or beyond). Overall, antibiotic therapy was modified once in 14 patients (35.0%), two to three times in 9 (22.5%, four to five times in 5 (12.5%), and more than five times in 3 (7.5%) (Figure 4).

#### 3.6.2. Corticosteroid Therapy

Due to prolonged inflammation, systemic corticosteroids were prescribed in 9 out of 40 patients (22.5%). Median hospital stay for steroid-treated patients was 28 days versus 18 days for those not receiving steroids; this difference was not statistically significant (*p* = 0.355).

#### 3.6.3. Oxygen Therapy and Respiratory Support

Oxygen therapy was required in 31 out of 40 patients (77.5%), with 17 receiving low-flow and 4 high-flow support. Six patients (15%) developed severe respiratory failure necessitating invasive or non-invasive ventilation in the Pediatric Intensive Care Unit (PICU), and in one case veno-venous extracorporeal membrane oxygenation (ECMO-VV). Among the six children requiring PICU admission, all presented with severe NP associated with CPPE. Etiologies included bacterial infections (*S. pyogenes*, *M. pneumoniae*) in combination with viral detections (RSV, SARS-CoV-2, Bocavirus and Influenza A).

#### 3.6.4. Surgical Management

Surgical intervention was required in cases with extensive pleural effusion, or hydropneumothorax. Twenty-four patients (66.7%) underwent pleural drainage when ultrasound findings were indicative of empyema (fibrin deposits, septations, etc.); 8 had isolated effusion, while 16 had both effusion and NP. Seven patients (17.5%) with severe parapneumonic effusion received intrapleural fibrinolytics (urokinase), five (12.5%) required thoracoscopic adhesiolysis after drainage failure, and one patient underwent lobectomy due to persistent necrotizing pneumonia in the context of acute myeloid leukemia.

### 3.7. Outcomes

No in-hospital mortality was observed. The median length of hospital stay was 18.5 days (IQR 16–26), and all patients were clinically stable at the time of discharge. Thirty patients (75.0%) continued oral antibiotics after discharge for a median duration of 8 days, (IQR 3.5–8), most commonly with cefpodoxime (21/30, 70.0%) or amoxicillin–clavulanate (7/30, 23.3%).

Post-discharge follow-up was completed in 38 of 40 patients (95.0%) and included clinical evaluation and imaging studies. Complete radiological resolution was documented in all patients at a median of 31 days following discharge (IQR 27–78). During follow-up, thirty-six patients (94.7%) required at least one chest X-ray to confirm complete radiological resolution, while four patients (10.5%) underwent at least one contrast-enhanced chest CT. Repeated CT scans were performed in cases of persistent pneumatocele, progressive hydropneumothorax, or for post-lobectomy assessment.

**Figure 5:**
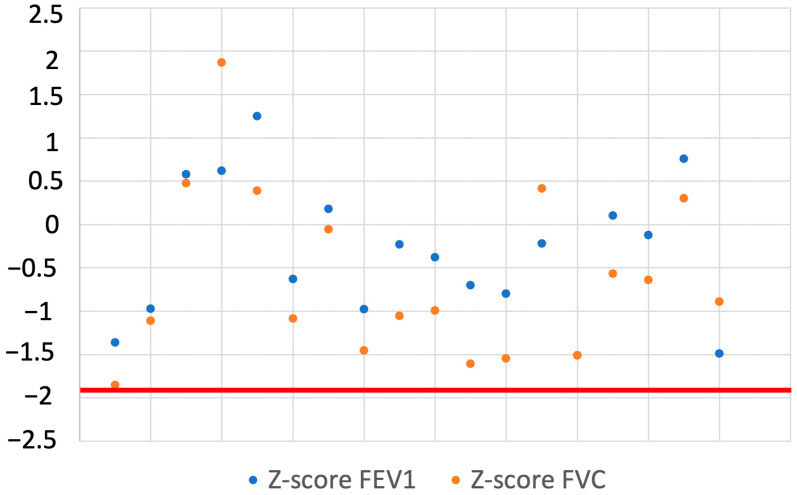
The scatter plot shows the Z-score values of FEV1 (blue dots) and FVC (orange dots) for each patient. The horizontal red line represents the lower threshold of the Z-score distribution (−1.96).

Long-term follow-up was available for 18 of 40 children (45.0%) at a median of 15 months (IQR 8–23) and included clinical assessment and spirometric evaluation. All reassessed patients were asymptomatic and had normal physical examinations. Spirometric measurements were within normal limits: mean Z-score FEV1 −0.305 ± 0.80, predicted FEV1 95.78% ± 9.97; mean Z-score FVC −0.943 ± 0.98, predicted FVC 92.58% ± 12.62 (Figure 5). Bronchodilator responsiveness testing was negative in all patients.

## 4. Discussion

This retrospective study describes a cohort of 40 children hospitalized with cCAP between January 2022 and September 2024 at the Department of Women’s and Children’s Health, University Hospital of Padua. Despite the substantial morbidity and the need for multidisciplinary management during the acute phase, overall outcomes were favorable both in the short and long term, with complete clinical and functional recovery (restitutio ad integrum) observed in the majority of patients.

The estimated incidence of cCAP among hospitalized pneumonia cases was 13.15%, which is higher than previously reported rates [21,22,23]. This finding likely reflects the tertiary referral role of our center, which receives the most complex cases from northeastern Italy, as evidenced by the fact that 65% of patients were transferred from other hospitals. Continued prospective data collection will enable future analyses to evaluate temporal trends in cCAP incidence and further contextualize these findings.

The mean age was 4.4 years, consistent with other reports indicating higher susceptibility in preschool-aged children [2,9]. Most patients were previously healthy and vaccinated according to regional immunization schedules, as previously described in existing literature [10]. The increased vulnerability of preschool children is likely due to immunological immaturity, frequent exposure to respiratory pathogens, and increased, less-regulated inflammatory responses [24,25].

Clinically, 67.5% presented with NP with CPEE, 22.5% with isolated CPEE, and 10% with NP without effusion, reflecting the frequent association between parenchymal necrosis secondary to vasculitis or intrapleural vessel thrombosis and empyema formation [11]. Similar findings were reported by Abdelhady et al. [9], with 54% of necrotizing pneumonia cases associated with effusion.

All patients were febrile at disease onset, with a median fever duration of 18 days (IQR 15–27), underscoring the high morbidity associated with cCAP. Persistent fever was accompanied by marked elevation of CRP and PCT (median peak value 300 mg/L and 14.39 µg/L, respectively). Notably, peak CRP and PCT did not temporally coincide, suggesting a dissociation between PCT, an early marker of bacterial infection, and CRP, which more closely reflects sustained cytokine activation and tissue injury. This discordance may be particularly relevant in necrotizing cCAP, where systemic inflammation can persist after adequate treatment of the primary infection, contributing to ongoing clinical severity. To our knowledge, such a comparative temporal assessment of CRP and PCT has not been previously been reported in pediatric cCAP.

From a microbiological perspective, positive pleural fluid or blood cultures were obtained in 30% of patients, a proportion slightly higher than that reported by Abdelhady et al. [9] (17–23%). This difference likely reflects the systematic collection of blood samples prior to antibiotic administration in our cohort. Bacterial–viral co-infection was observed in 10% of cases, supporting the hypothesis that viral infections may predispose the respiratory epithelium to subsequent bacterial invasion through impaired mucociliary clearance and upregulation of cellular receptors [26]. *Streptococcus pneumoniae* was the most frequently identified pathogen, followed by *Streptococcus pyogenes*, in contrast to other studies in which *Staphylococcus aureus* is reported as the second most common etiological agent [9,10,11]. Notably, blood cultures were positive in only 17.5% of cases, with *S. pneumoniae* identified in five patients, despite all children being fully vaccinated according to the national immunization schedule, including PCV13. The persistence of *S. pneumoniae* as a leading pathogen suggests that pneumococcal cCAP can still occur in vaccinated children, possibly due to infection with non-vaccine serotypes. Ongoing and future data collection will help clarify potential temporal trends in microbiological patterns, which remain critical for guiding targeted antibiotic therapy.

Regarding diagnostic strategies, contrast-enhanced chest CT continues to represent the gold standard for the diagnosis of NP. In our cohort, imaging modalities involving ionizing radiation were frequently employed, with a median of five chest X-rays per patient and at least one CT scan performed in 85% of cases. Thoracic ultrasound is increasingly recognized as a valuable tool for both diagnosis and follow-up [6,27], offering high diagnostic accuracy while minimizing radiation exposure. In our center, ultrasound was employed for monitoring pleural effusion, whereas diagnostic evaluation relied exclusively on CT imaging or chest X-ray. Beyond its diagnostic role, lung ultrasound provides important practical advantages in the therapeutic management of these patients, allowing real-time bedside assessment of pleural effusions, consolidations, and dynamic changes during treatment [6]. Its repeatability and safety make it particularly useful for monitoring disease progression and response to therapy, supporting clinical decision-making regarding antibiotic modification, drainage procedures, and timing of further imaging. Moreover, lung ultrasound can be readily integrated into routine clinical practice, even in critically ill children, reducing the need for repeated radiographic or CT examinations [6]. For these reasons, broader integration of thoracic ultrasound into diagnostic and management protocols for pediatric cCAP should be encouraged.

Antibiotic therapy represented the cornerstone of medical management, with frequent adjustments during hospitalization. Only 22.5% of patients remained on the initial antibiotic regimen, whereas 77.5% required at least one modification, a finding consistent with previous reports. Greater awareness that many clinical manifestations of cCAP are predominantly driven by inflammatory rather than ongoing infectious processes may help reducing unnecessary antibiotic escalation and repeated thoracic imaging.

In our cohort, systemic corticosteroids were administered in 22.5% of patients, based on an individualized clinical decision-making process based on the evidence of persistent systemic inflammation. The use of corticosteroids in cCAP, including NP, is primarily aimed at modulating an exaggerated inflammatory response that contributes to parenchymal lung injury and pleural complications [28,29,30,31]. Although some evidence suggests that corticosteroids may reduce inflammatory markers, fever duration, and short-term morbidity in severe pediatric pneumonia, the available data remain limited and heterogeneous [28,29,30,31]. Current international guidelines do not recommend the routine use of corticosteroids in pediatric NP but acknowledge a potential role in selected cases with severe disease and hyperinflammatory features [31]. Therefore, further prospective and randomized studies are needed to better define the efficacy, optimal dosing, and safety profile of systemic corticosteroids in pediatric cCAP, as well as to identify clinical or biological predictors of response.

Overall outcomes were favorable. Complete radiological resolution was achieved at a median of 31 days following discharge. Long-term follow-up, conducted at a median of 15 months, demonstrated that all reassessed patients were asymptomatic and exhibited normal physical examination results and spirometric findings. These results confirm that, despite severe initial presentation and prolonged course, the majority of children experience complete recovery after cCAP.

### Study Limitations

This study has several limitations. Its retrospective design may introduce selection and information bias, and follow-up data were incomplete for some patients, particularly those transferred to other centers after the acute phase of the disease. Incidence comparisons with pre-2022 periods were not feasible due to lack of historical data. Microbiological investigations were not fully standardized in terms of tests performed or their timing relative to disease onset, resulting in heterogeneous data, and pneumococcal serotyping was not available precluding definitive conclusions regarding vaccine escape strains. In addition, spirometry was performed in only in a minority of patients, mainly due to the age-related limitations. Finally, the relatively small cohort size represents a significant limitation; however, this reflects the real-life nature of this single-center, retrospective study and the relative rarity of hospitalized pediatric cCAP.

## 5. Conclusions

This retrospective cohort of 40 children with cCAP, from a biochemical perspective, exhibited elevated CRP and PCT levels, with a temporal dissociation of peak values, suggesting persistent systemic inflammation even after control of the primary infection. Despite the significant clinical impact of cCAP in terms of morbidity and the need for multidisciplinary management during the acute episode, the collected data confirm generally favourable outcomes in both the short and long term, with restitutio ad integrum in most patients. A clearer understanding of the predominantly inflammatory, rather than infectious, nature of the condition may help reduce unnecessary interventions and multiple antibiotic changes. Nevertheless, ongoing data collection and further prospective studies are warranted to optimize clinical, microbiological, and instrumental management of pediatric cCAP, as well as to identify predictive factors of disease severity and tailor therapeutic interventions.

## Data Availability

The raw data supporting the conclusions of this article are not available due to privacy reasons.
